# Revealing the Phenolic Acids in *Cardamine violifolia* Leaves by Transcriptome and Metabolome Analyses

**DOI:** 10.3390/metabo12111024

**Published:** 2022-10-26

**Authors:** Shen Rao, Xin Cong, Haodong Liu, Yili Hu, Wei Yang, Hua Cheng, Shuiyuan Cheng, Yue Zhang

**Affiliations:** 1 School of Modern Industry for Selenium Science and Engineering, National R&D Center for Se-Rich Agricultural Products Processing Technology, Wuhan Polytechnic University, Wuhan 430023, China; 2 Enshi Se-Run Material Engineering Technology Co., Ltd., Enshi 445000, China

**Keywords:** *Cardamine violifolia*, leaf, metabolome, phenolic acids, transcriptome

## Abstract

*Cardamine violifolia*, a species belonging to the Brassicaceae family, is a selenium hyperaccumulator and a nutritious leafy vegetable. Our previous study showed that *C. violifolia* leaves are rich in total phenolic acids, but the composition and corresponding genes remain unknown. In this study, we investigated the phenolic acid compounds and potential gene regulation network in the outer leaves (OL) and central leaves (CL) of *C. violifolia* using transcriptome and metabolome analyses. Results showed that the OL contained a higher total phenolic acid content than the CL. Metabolome analysis revealed a total of 115 phenolic acids, 62 of which (e.g., arbutin, rosmarinic acid, hydroxytyrosol acetate, and sinapic acid) were differentially accumulated between the CL and OL of *C. violifolia*. Transcriptome analysis showed that the differentially expressed genes were significantly enriched in the pathways of secondary metabolite biosynthesis and phenylpropanoid biosynthesis. Conjoint analysis of the transcriptome and metabolome indicated that seven genes (*CYP84A1*, *CYP84A4*, *CADH9*, *SGT1*, *UGT72E1*, *OMT1*, and *CCR2*) and eight phenolic acids (sinapic acid, sinapyl alcohol, 5-O-caffeoylshikimic acid, sinapoyl malate, coniferin, coniferyl alcohol, L-phenylalanine, and ferulic acid) constituted a possible regulatory network. This study revealed the phenolic acid compounds and possible regulatory network of *C. violifolia* leaves and deepened our understanding of its nutrient value.

## 1. Introduction

*Cardamine violifolia*, a popular leafy vegetable crop belonging to the Brassicaceae family, is native to Enshi, Hubei, China, widely cultivated in the Hubei province of China, and well-known for its ability to accumulate selenium [1]. Our previous studies demonstrated that *C. violifolia* can accumulate extremely high levels of selenium in its leaves and roots [2,3]. Selenium in *C. violifolia* has been mainly found in organic forms [3]; however, the dominant organic selenium species are disputable. A recent study found that the major form of selenium in *C. violifolia* is selenolanthionine [4]. Other researchers suggested that *C. violifolia* primarily accumulates selenocystine [1,5]. The controversy regarding the main form of organic selenium in *C. violifolia* requires further investigation. The predominant form of selenium appears to be selenolanthionine because its detection method is relatively advanced and extensive.

Phenolic acids are a class of secondary metabolites widely present in plants. They function in response to biotic or abiotic stress in plants and are closely related to the color, flavor, and taste of vegetables and fruits [6]. Phenolic acids have strong antioxidant capacities and are involved in various browning and redox reactions in plants [7]. These compounds generally contain one carboxylic acid group and primarily consist of two sub-classes: hydroxybenzoic and hydroxycinnamic acid types [8]. Phenolic acids are usually biosynthesized through the shikimic acid and phenylpropanoid metabolic pathways in plants [9]. Gallic acid is the most common phenolic acid derived from the shikimic acid pathway [10]. Most phenolic acid compounds, such as caffeic acid, ferulic acid, sinapic acid, and p-coumaric acid, are biosynthesized through the phenylpropanoid pathway [11]. Phenylalanine ammonia-lyase (PAL), 4-coumarate-CoA ligase (4CL), and cinnamate-4-hydroxylase (C4H) are key enzymes involved in the phenylpropanoid pathway [12]. PAL and C4H participate in the transformation of phenylalanine into several phenolic acids (e.g., trans-cinnamic acid, benzoic acid, salicylic acid, p-coumaric acid, caffeic acid, ferulic acid, and sinapic acid) [13,14,15]. These phenolic acids can be converted into coumarin, chlorogenic acid, and caffeic acid under the catalysis of 4CL and further metabolized through the flavonoid metabolism pathway [16,17].

As important bioactive compounds, phenolic acids have attracted increasing attention because of their remarkable capacity for free radical scavenging, antioxidant, anti-inflammatory, anticancer, and bacteriostatic properties in humans [8,18]. The daily intake of phenolic acids ranges from 25 mg to 1 g in humans, depending on the type and quality of food [19]. Vegetables of the Brassicaceae family are rich in phenolic acids. For example, the total phenolic content in *Brassica fruticulosa* subsp. *fruticulosa* is 32.63 ± 1.11 mg gallic acid equivalent in per gram of leaf extract, and sinapic and ferulic hydroxycinnamic acids are the most abundant phenolic acids [20]. High levels of phenolic acids, such as hydroxycinnamic acid, caffeic acid, salicylic acid, 4-coumaric acid, and ferulic acid are found in three Brassica crops, namely, Chinese cabbage, white cabbage, and kale [21]. As a member of the Brassicaceae family, *C. violifolia* also has rich nutrients and a delicious taste, apart from its strong capacity to accumulate selenium [22]. Our previous study revealed that *C. violifolia* is rich in phenolic acids, flavonoids, and glucosinolates [22]. However, only a few works have focused on these nutrient compounds.

Even though the biosynthetic route has been identified [9], phenolic chemicals in diverse plants are complicated. The phenolic acids in *C. violifolia* remain unclear and may have distinctive characteristics. This study aimed to identify the phenolic acids in the leaves of *C. violifolia*. The rosette leaves were divided into outer leaves (OL) and central leaves (CL). The phenolic acids in the leaves were detected using widely targeted metabolome technology. Transcriptome sequencing was performed to identify the key genes that regulate the accumulation of phenolic acids in *C. violifolia* leaves. This study is the first to focus on the phenolic acids in *C. violifolia*. The discovered phenolic acids and key potential genes will help understand the nutritional value of *C. violifolia* and promote its further consumption as a novel vegetable.

## 2. Materials and Methods

### 2.1. Plant Materials

**Figure 1:**
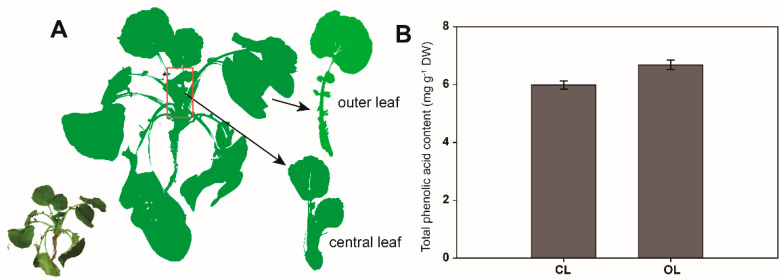
Schematic diagram of sampling (**A**) and total phenolic acid content (**B**) in *C. violifolia* leaves.

Given that *C. violifolia* is native to Enshi with a large cultivation scale, it is representative to sample it from the local area. On 24 November 2021, two-year-old *C. violifolia* plants were harvested at the cultivation farm of Enshi Se-run Material Engineering Technology Co., Ltd. (Enshi, China), in Erpo Village (30°21′48″ N, 109°33′36″ E, altitude 500 m above sea level, average temperature 16/10 °C of day/night in November), Longfeng Town, Enshi City, Hubei Province, China. The leaves of the plants were collected separately as CL and OL. For leaf growth, the three newest leaves were designated as CL, and the remaining leaves were labeled as OL (Figure 1A). The CL and OL collected from 20 plants were frozen in liquid nitrogen, crushed, and divided into four parts. Each part was separately stored to prepare four biological replicates. The samples were then stored at −80 °C.

### 2.2. Measurement of Total Phenolic Acid Content

Total phenolic acid content was determined following the method described by Rao et al. (2021) [22]. Briefly, samples (0.5 g) were weighed, added with 10 mL of 50% methyl alcohol, and incubated in an ultrasonic oscillator at 60 °C for 30 min. The supernatants were collected after centrifugation at 10,000 rpm for 15 min and reacted with Folin–Ciocalteu reagent. The phenolic acid content of each sample was determined using a spectrophotometer at 765 nm. Gallic acid was used to generate the standard curve.

### 2.3. Widely Targeted Metabolome Detection and Analysis

The samples were freeze-dried by a vacuum freeze-dryer and crushed into powder by a mixer mill. The lyophilized powder (100 mg) was mixed with 1.2 mL of 70% methanol, blended by a vortex six times, and rested overnight at 4 °C. The samples were then centrifuged at 12,000 rpm for 10 min. The supernatants were filtered (0.2 μm, Anpel, Shanghai, China) for ultra-high performance liquid chromatograph-mass/mass (UPLC-MS/MS) analysis. The metabolites were detected by the UPLC-MS/MS (UPLC: Shimadzu Nexera X2; MS: Applied Biosystems 4500 Q TRAP) at Metware Bio-Tech. Co. (Wuhan, China). Hierarchical cluster analysis and Pearson correlation analysis were carried out by the R package, ComplexHeatma. OPLS-DA patterns were constructed by using the R package, MetaboAnalystR. The VIP values were extracted from the OPLS-DA results. Differentially regulated metabolites between groups were determined by VIP ≥ 1 and absolute log_2_fold change ≥ 1. The metabolites were annotated in the KEGG compound database and then mapped to KEGG pathways. Their significance was determined by hypergeometric test *p*-values.

### 2.4. Transcriptome Sequencing and Analysis

Total RNA was extracted using RNApre Pure Plant Kit (Tiangen Biotech, Beijing, China). The purity, concentration, and integrity of the total RNA were respectively measured to ensure RNA quality. One microgram of total RNA from each sample and NEBNext^®^ UltraTM RNALibrary Prep Kit for Illuminawere (NEB, Ipswich, MA, USA) was used for construction of sequencing libraries, following the operation manual. The cDNA sequences were purified and qualitatively evaluated by the Agilent Bioanalyzer 2100 system (Agilent Technologies Inc., Palo Alto, CA, USA). The cDNA libraries were then sequenced by an Illumina Novaseq 600 system at Metware Bio-Tech. Co. (Wuhan, China). Clean reads were obtained after removing adapters, high-N content, and low-quality reads. De novo assembly of the transcriptome was performed using Trinity (v2.11.0). Corset was used to regroup relevant transcripts into ‘gene’ clusters (https://github.com/trinityrnaseq/trinityrnaseq (accessed on 20 December 2021). The candidate coding regions of the genes were identified using TransDecoder r (https://github.com/TransDecoder/TransDecoder/wiki (accessed on 21 December 2021)). Gene function was annotated based on the following databases: Nr (NCBI non-redundant protein sequences); Swiss-Prot (a manually annotated and reviewed protein sequence database); Trembl (a variety of new documentation files and the creation of TrEMBL, a computer annotated supplement to SWISS-PROT); KEGG (Kyoto Encyclopedia of Genes and Genomes); GO (gene ontology); KOG/COG (COG: clusters of orthologous groups of proteins; KOG: euKaryotic Ortholog Groups); Pfam (protein family). Gene expression levels were calculated by RSEM software and presented as fragments per kilobase of exon model per million mapped fragments (FPKM). Differences between groups were analyzed by DESeq2 (v1.22.1). Significance between groups was set at *p* ≤ 0.05 and absolute log_2_fold change ≥ 1. Differentially expressed genes (DEGs) were performed with KEGG and GO enrichment analysis.

### 2.5. Conjoint Analysis of Transcriptome and Metabolome

Conjoint analysis of transcriptomes and metabolomes was carried out to reveal the integrative correlation between phenolic acids and genes. These results would help us comprehensively understand the changes in phenolic acid content in *C. violifolia* leaves. The DEGs and DRMs were synchronously mapped to KEGG pathways. The co-enriched KEGG pathways of DEGs and DRMs were analyzed based on *p*-values. Correlation analysis of the DRMs and DEGs was performed using the Cor function in R. The DRMs and DEGs were screened to construct a network using the threshold of Pearson’s correlation coefficient ≥0.9 and *p* ≤ 0.05.

### 2.6. Real-Time Quantitative PCR (RT-qPCR) Validation

To validate the accuracy of the expression levels evaluated by the transcriptome, RT-qPCR was performed using the LineGene 9600 Plus Fluorescent Quantitative PCR System (Bioer, Hangzhou, China). The synthesis of the first-strand cDNA and fluorescent detection were carried out using the Real Time One Step RT-qPCR kit (FP313, SYBR Green, Tiangen Biotech, Beijing, China). The primers were designed using Primer3plus (http://www.primer3plus.com/cgi-bin/dev/primer3plus.cgi (accessed on 5 May 2022). The primer sequences are listed in Table S1. The relative expression of a gene in a given sample was calculated using the formula F = 2^−ΔΔCt^. The reference gene, β-actin3, was selected to normalize the relative expression levels of the genes. Four biological replicates were set in the RT-qPCR. Each sample was analyzed with three technical replicates.

### 2.7. Statistical Analysis

All data are presented as mean values representing four biological triplicates ± standard errors. Data were analyzed by one-way ANOVA in SPSS22 (SPSS Inc., Chicago, IL, USA). Multiple treatment groups were compared by Duncan’s honestly significant difference test at *p* ≤ 0.05.

## 3. Results

### 3.1. Content of Total Phenolic Acids

The total phenolic acid content of the OL (6.68 mg g^−1^ DW) was higher than that of the CL (5.99 mg g^−1^ DW) of *C. violifolia* (Figure 1B). This result indicated that the phenolic acid content changes with the growth of *C. violifolia* leaves.

### 3.2. Overview of the Metabolome Detection

**Figure 2:**
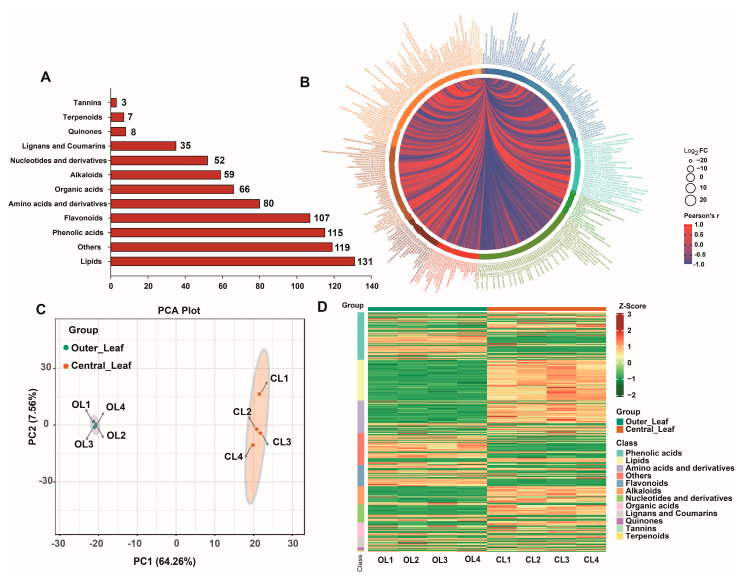
Overview of the metabolites: (**A**) categorical statistics of the metabolites; (**B**) correlations between the metabolites; (**C**) principal component analysis of the samples; and (**D**) clustering heat map of the metabolites.

A widely targeted metabolome analysis was carried out to reveal changes in metabolites between CL and OL, especially in phenolic acid compounds. A total of 782 metabolites were detected in the OL and CL of *C. violifolia*. The metabolites were classified into 12 subcategories, including lipids, phenolic acids, flavonoids, amino acids and derivatives, alkaloids, and terpenoids. Lipids, phenolic acids, and flavonoids were the dominant metabolites with 131, 115, and 107 members (Figure 2A), respectively. All the detected metabolites were clustered and subjected to correlation analysis. Significant correlations were observed between the metabolites (Figure 2B). Principal component analysis (PCA) was conducted based on the relative content of the metabolites in the samples. The results showed discrimination between the OL and CL samples, but the metabolites were clustered within groups (Figure 2C), indicating that the samples from the same *C. violifolia* leaves had good repeatability. The metabolites were further clustered and normalized to reveal the changes between the OL and CL samples. As shown in Figure 2D, remarkable changes in the content of phenolic acids, lipids, and amino acids were observed between the OL and CL samples.

### 3.3. Analysis of Phenolic Acids

**Figure 3:**
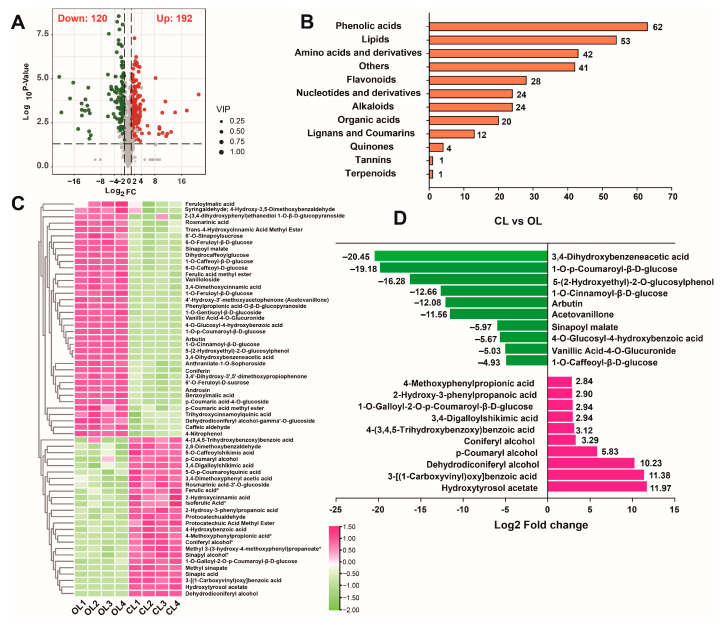
Analysis of the DRMs: (**A**) statistics of down- and upregulated metabolites between the CL and OL; (**B**) categorical statistics of the DRMs; (**C**) clustering heatmap of the differentially regulated phenolic acids; and (**D**) top 20 changed phenolic acids.

DRMs were screened between the OL and CL groups using the threshold of fold changes and *p*-values. A total of 312 metabolites, including 192 upregulated and 120 downregulated metabolites, exhibited significantly altered contents in the CL and OL (Figure 3A). The content profiles of all DRMs showed that most DRMs accumulated more in the CL than in the OL (Figure S1A). KEGG pathway enrichment of the DRMs showed that they were significantly enriched in several pathways, including the metabolic pathway, biosynthesis of secondary metabolites pathway, and phenylpropanoid biosynthesis pathways (Figure S1B). The DRMs were further classified into 12 subcategories, depending on their chemical structures. The results revealed that phenolic acids accounted for most of the members, followed by lipids and amino acids and derivatives with 62, 53, and 42 members (Figure 3B), respectively. These results indicated that phenolic acids were the main differential metabolites between the CL and OL of *C. violifolia*. The relative content profiles of the phenolic acids from the DRMs showed that 37 phenolic acid compounds (such as arbutin, feruloylmalic acid, rosmarinic acid, and sinapoyl malate) had higher contents in the OL than in the CL, and 25 phenolic acids (including hydroxytyrosol acetate, ferulic acid, coniferyl alcohol, and sinapic acid) accumulated more in the CL than in the OL, (Figure 3C). The 20 phenolic acid compounds with the greatest content change, including 10 that were higher in OL and 10 that were higher in the CL, were analyzed to reveal the differences in phenolic acids in the OL and CL samples (Figure 3D). The results showed that the contents of 3,4-dihydroxybenzeneacetic acid, 1-O-p-coumaroyl-β-D-glucose, 5-(2-hydroxyethyl)-2-O-glucosylphenol, 1-O-cinnamoyl-β-D-glucose, arbutin, and acetovanillone were substantially lower in the CL than in the OL, and the contents of hydroxytyrasol acetate, 3-[(1-carboxyvinyl)oxy]benzoic acid, and dehydrodiconiferyl alcohol were higher in the CL than in the OL (Figure 3D).

### 3.4. Transcriptome Sequencing and Annotation

Transcriptome sequencing generated 46,767,574–53,105,006 raw reads from the eight OL and CL samples. After the adapters and low-quality reads were removed, a total of 45,093,828–50,010,102 clean reads were obtained with Q30 values higher than 92% in each sample library. The data volume in each library was greater than 6 Gb. The clean reads were then spliced and assembled using Trinity, resulting in a total of 119,939 unigenes with an average length of 1241 bp. Finally, the unigenes were mapped to seven databases. The results showed that 60,173, 82,706, 62,987, 82,521, 50,137, 72,691, and 57,208 unigenes were annotated using the KEGG, Nr, SwissProt, TrEMBL, KOG, GO, and Pfam databases, respectively (Table 1). A total of 84,140 unigenes were annotated in at least one of the seven databases.

### 3.5. Analysis of DEGs

**Figure 4:**
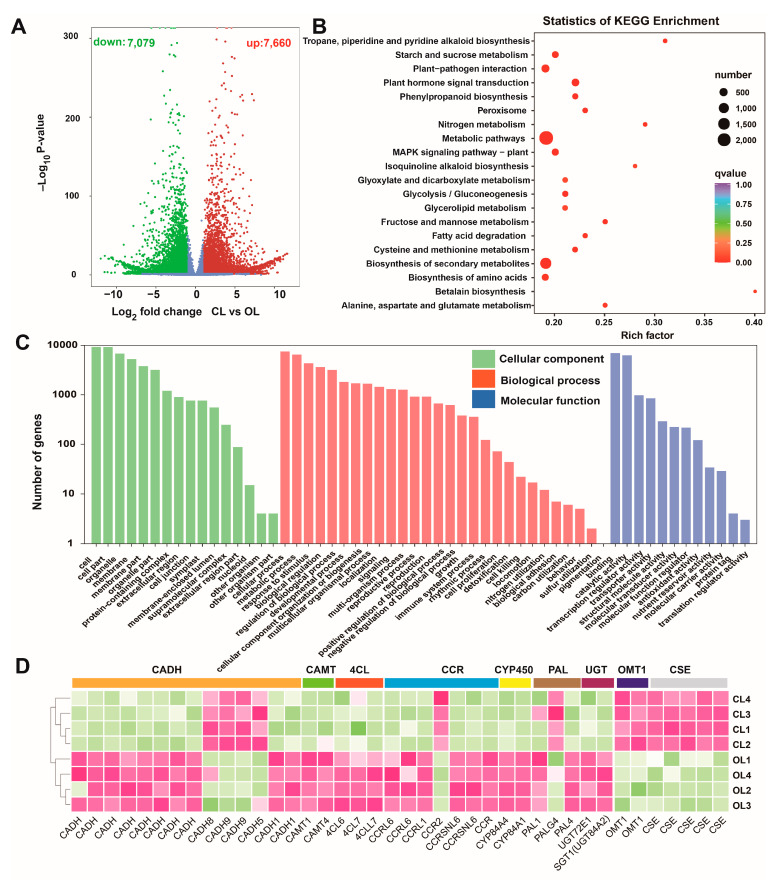
Analysis of the DEGs: (**A**) volcano diagram of the DEGs; (**B**) KEGG pathway enrichment of the DEGs; (**C**) GO enrichment of the DEGs; and (**D**) expression profiles of the DEGs involved in the phenylpropanoid biosynthesis pathway.

The biological repeatability between the samples was estimated using Pearson’s correlation coefficient. The results showed that the samples with inner CL or OL had good biological repeatability, but poor correlations were found between the groups (Figure S2A). The differences between the OL and CL samples were evaluated via PCA, based on the FPKM values of the unigenes (Figure S2B). The findings indicated that the expression levels of the unigenes remarkably varied between the OL and CL. The DEGs were screened by setting a threshold. A total of 14,739 DEGs, including 7079 downregulated and 7660 upregulated DEGs, were obtained in the comparison of CL and OL (Figure 4A). Analysis of KOG classification, KEGG pathway, and GO term enrichment of the global DEGs was performed. The KOG classification of the DEGs showed that general function prediction, posttranslational modification, protein turnover, chaperones, and signal transduction mechanisms were the largest subclasses (Figure S3). The DEGs were significantly enriched in several KEGG pathways, such as metabolic pathways (Ko01100), biosynthesis of secondary metabolites (Ko01100), and phenylpropanoid biosynthesis (Ko00940) (Figure 4B). The pathways of biosynthesis of secondary metabolites and phenylpropanoid biosynthesis contained 1331 and 164 members, respectively. GO enrichment analysis revealed that the largest subcategories were cells, cell parts, organelles, membranes, and membrane parts for cellular components; cellular process, metabolic process, response to stimulus, biological regulation, and regulation of biological process for biological processes; and binding and catalytic activity for molecular function (Figure 4C). DEGs in the phenylpropanoid biosynthesis pathway were also analyzed and enzyme genes related to phenolic acid biosynthesis were extracted. As shown in Figure 4D, 40 DEGs clustered into nine classes, namely, *cinnamyl-alcohol dehydrogenase* (*CADH*), *caffeoyl-CoA O-methyltransferas* (*CAMT*), *4-coumarate—CoA ligase* (*4CL*), and *cinnamoyl-CoA reductase* (*CCR*), *cytochrome P450* (*CYP450*), *phenylalanine ammonia-lyase* (*PAL*), *UDP-glycosyltransferase* (*UGT*), *flavone 3’-O-methyltransferase 1* (*OMT1*), and caffeoylshikimate esterase (*CSE*) were screened. Their expression profiles showed that most of them had higher expression levels in the OL than in the CL. However, several genes, including *CADH8*, *CADH9*, *CCR2*, *OMT1*, and *CSE*, had higher transcription levels in the CL than in the OL.

### 3.6. Conjoint Analysis of Transcriptome and Metabolome

**Figure 5:**
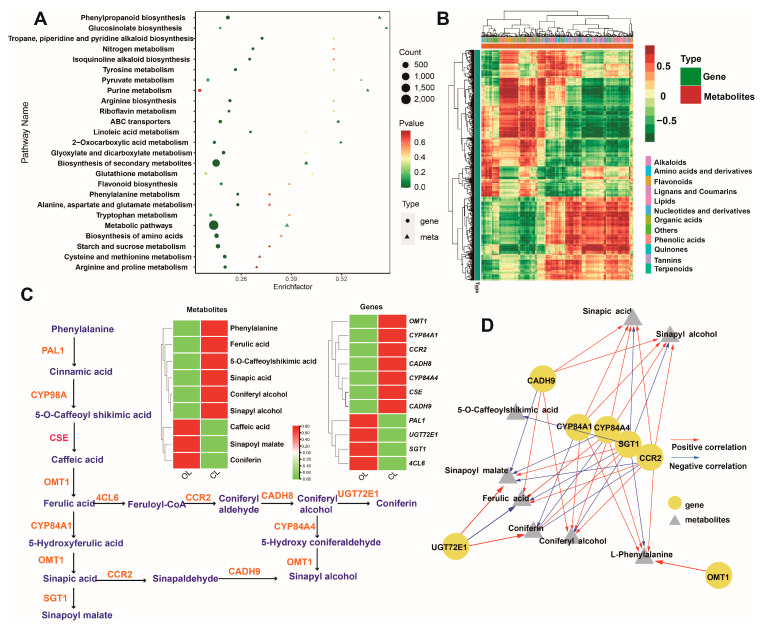
Conjoint analysis of the DEGs and DRMs: (**A**) KEGG pathways enrichment of the DRMs and DEGs; (**B**) clustering heatmap of the DEGs and DRMs; (**C**) DEGs and phenolic acids in the phenylpropanoid biosynthesis pathway; and (**D**) potential network between the genes and phenolic acids.

A conjoint analysis of the transcriptomes and metabolomes was performed to reveal the possible correlation between the DEGs and DRMs involved in the biosynthesis of phenolic acids. The DRMs and DEGs were jointly mapped to KEGG pathways. The result showed that the DRMs and DEGs were significantly co-enriched in the pathways of phenylpropanoid biosynthesis and biosynthesis of secondary metabolites (Figure 5A). The correlation heatmap of the DRMs and DEGs showed that numerous metabolites and genes, including phenolic acids and some genes, were significantly correlated (Figure 5B). Therefore, the phenolic acids and genes involved in the phenylpropanoid biosynthesis pathway were further analyzed. DRMs that were annotated as phenolic acids and genes in the phenylpropanoid biosynthesis pathway were extracted and a biosynthesis pathway for the phenolic acids and their corresponding genes was established. As shown in Figure 5C, 9 phenolic acids and 11 genes in the pathway had different contents or expression levels in the CL and OL of *C. violifolia*. For example, sinapyl alcohol content and catalytic enzyme gene *OMT1* expression were higher in the CL than OL and caniferin content and corresponding enzyme gene *UGT72E1* expression were higher in the OL than in the NL. The correlations between the DRMs and DEGs involved in the biosynthesis of phenolic acids were further analyzed. The results showed that seven genes and eight phenolic acids constituted a possible regulatory network (Figure 5D). For instance, *CYP84A1* showed a positive correlation with four phenolic acids, namely, sinapic acid, sinapyl alcohol, coniferyl alcohol, and phenylalanine, and a negative correlation with coniferin. *CCR2* was positively correlated with sinapic acid, sinapyl alcohol, ferulic acid, coniferyl alcohol, and phenylalanine, but negatively correlated with sinapoyl malate and coniferin.

### 3.7. Correlation between Transcriptome and Real-Time PCR (RT-qPCR) Results

**Figure 6:**
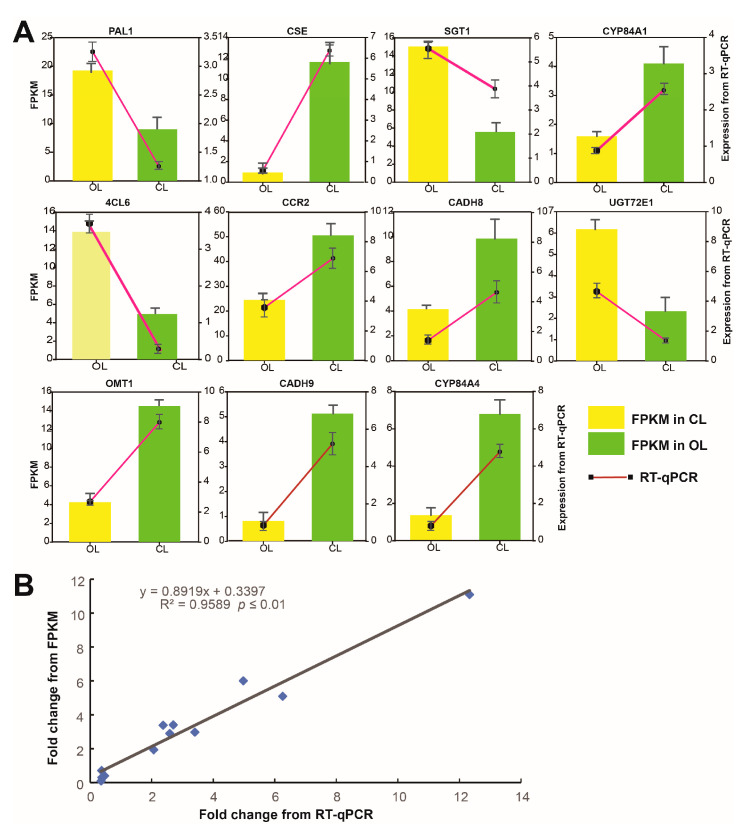
Analysis of the RT-qPCR results: (**A**) expression levels of the selected genes and (**B**) integrative correlation between the RT-qPCR and transcriptome results.

RT-qPCR was conducted on the 11 genes involved in the biosynthesis of phenolic acids to validate the accuracy of the transcriptomic data. The results showed that the relative expression levels from RT-qPCR exhibited similar changing trends to the FPKM values of the 11 genes (Figure 6A). Integrative correlation analysis revealed that the data generated from the RT-qPCR and transcriptome were significantly correlated (Figure 6B), indicating that the transcriptome data were credible.

## 4. Discussion

Phenolic acids are important secondary metabolites in plants and exhibit beneficial effects on human health, such as scavenging free radicals, preventing cardiovascular diseases, protecting against cancer, and relieving neurodegenerative diseases. [23]. In recent years, plant phenolic acids have attracted increasing attention because of their health benefits. Phenolic acid compounds have been found in several Brassicaceae crops, such as broccoli [24] and cabbage [25]. As a member of the Brassicaceae family, *C. violifolia* is also rich in phenolic acids; however, minimal information is available on the phenolic acids in *C. violifolia*. As the first to focus on the phenolic acid in *C. violifolia*, this study enriched our knowledge of this species.

The total phenolic acid content in a crop varies with its development. In addition, the changing trend differs for each species. For example, total phenolic content decreases in navel oranges during fruit maturation [26]. In contrast, the content of total phenolic acids and several individual phenolic compounds, such as quercetin-3-O-gentiobioside and isoquercitrin, increase in okra fruits at 4–6 days post-anthesis and then decreases at 7–9 days post-anthesis [27]. In the present work, total phenolic acid content was higher in the OL than in the CL. Our previous study also revealed that total phenolic acids accumulate more in *C. violifolia* leaves at the podding stage than those at the rosette stage, even under selenate treatment [22]. These results indicated that phenolic acids tend to accumulate during the growth of *C. violifolia* leaves. The present study is the first to identify the phenolic acid compounds in *C. violifolia*. A total of 115 phenolic acids were identified. Several common phenolic acids, such as chlorogenic, caffeic, sinapic, and ferulic acids, have also been found in broccoli [24] and cabbage [25]. Therefore, plants in the Brassicaceae family share the same phenolic compounds. For example, benzoylmalic acid (also known as malic acid benzoate) was found in *C. violifolia* leaves and has also been isolated from *Lepidium meyenii* Walpers, a member of the Brassicaceae family [28]. Although the function of benzoylmalic acid in plants and humans is still unclear, a recent study showed that it may protect peach aphids against ultraviolet radiation [29].

This study revealed the DRMs between CL and OL and showed the top 10 differential phenolic acids. These results provided insights into the changes in phenolic acids with the development of *C. violifolia* leaves and indicated that *C. violifolia* leaves differentially accumulate phenolic acids in different tissues. For instance, arbutin is a phenolic glucoside widely present in various plants, such as tea, peaches, and coffee [30], and has various health benefits for humans, such as scavenging free radicals [31], diminishing inflammation [32] and hyperpigmentation [33], and even protecting the liver against alcohol-induced injury [34]. Moreover, arbutin shows high safety and does not easily trigger unnecessary health issues, such as irritation and toxicity [35]. This work found that arbutin was more accumulated in the OL of *C. violifolia* than in the CL, implying that the OL are suitable for arbutin extraction. A recent study pointed out that *C. violifolia* powder, mainly from old leaves, had excellent antioxidant effects in weaned pigs [36]. Arbutin possibly plays a role in this effect. In contrast, hydroxytyrosol acetate has significantly greater content in the CL than in the OL, indicating that new leaves of *C. violifolia* tend to accumulate this phenolic compound. Hydroxytyrosol acetate is a phenolic compound that has been found in olives [37], and exhibits functions such as oxidation resistance, heart protection, and antibacterial activity [37,38]. Owing to its health benefits, hydroxytyrosol acetate has been widely applied as functional food additions and nutraceuticals [38]. Therefore, the CL of *C. violifolia* may be a novel source of natural hydroxytyrosol acetate. However, the absolute hydroxytyrosol acetate content in *C. violifolia* requires further investigation.

The conjoint analysis of transcriptomes and metabolomes revealed that the DEGs and DRMs were significantly enriched in the pathways of phenylpropanoid biosynthesis and secondary metabolite biosynthesis. The differential expression of the genes (e.g., *CYP84A1* and *PAL1*) involved in these pathways (Figure 5C) may greatly contribute to the differential accumulation of individual phenolic compounds (e.g., ferulic acid and sinapol malate) in the CL and OL of *C. violifolia*. The regulatory network formed by the seven candidate key genes and eight phenolic acids indicated the key genes and phenolic compounds that differed between the CL and OL (Figure 5D). These candidate genes encode the enzymes involved in the biosynthesis of downstream phenolic compounds and may also be correlated with other phenolic acids in the phenylpropanoid biosynthesis pathway. CYP84A1 and CYP84A4 are members of the cytochrome P450-dependent mono-oxygenase CYP84 subfamily [39]. CYP84, also known as ferulate-5-hydroxylase (F5H), catalyzes the conversion of coniferyl alcohol, coniferaldehyde, and ferulic acid to sinapic acid and syringyl lignin monomers [40].Significant correlations were observed between the two CYP84 genes (*CYP84A1* and *CYP84A4*) and several phenolic compounds (e.g., sinapic acid, coniferyl alcohol, sinapyl alcohol, and ferulic acid), implying that *CYP84A1* and *CYP84A4* may be involved in the biosynthesis or accumulation of these compounds in *C. violifolia*. However, a previous study showed that F5H1 (CYP84A1) is an indispensable enzyme for the expression of the genes involved in anthocyanin biosynthesis and accumulation [41]. CYP84A4 is a paralog of CYP84A1, which is specific to Arabidopsis and participates in the biosynthesis of arabidopyrones [42]. Therefore, the functionality of *CYP84A1* and *CYP84A4* in *C. violifolia* requires further investigation. CCR is a key enzyme that catalyzes the first step in monolignol biosynthesis and regulates lignin synthesis via the phenylpropanoid biosynthesis pathway [43]. Overexpression of CCR2 increases the content of lignin and resistance to *Sclerotinia sclerotiorum* in *Brassica napus* [44]. The present study indicated that CCR2 may be involved in the biosynthesis of several phenolic acids. Although this result enriches our understanding of the potential function of CCR2 in *C. violifolia*, further validation is necessary.

In summary, discrepancies in the amounts of individual phenolic acids accumulated in the CL and OL of *C. violifolia* contributed to variances in total phenolic acid content. Differences in the accumulation of the individual phenolic acids may be attributed to the expression changes of some key genes involved in the phenylpropanoid biosynthesis pathway. The expression levels of these genes change with the stage of development of *C. violifolia* leaves. Specifically, several key genes involved in the phenylpropanoid biosynthesis pathway altered their expression levels between the OL and CL, such as *CYP84A1*, *CYP84A4*, and *CCR2*. These genes may regulate the biosynthesis and accumulation of phenolic acids in *C. violifolia*. However, the regulatory network remains unclear and the genes identified in the current study may be candidates for further investigation of the biosynthesis mechanism of various phenolic acids in *C. violifolia*.

## 5. Conclusions

*C. violifolia* leaves are rich in phenolic acids. The OL contained a higher total phenolic acid content than the CL, indicating that phenolic acids tend to accumulate with the develop of *C. violifolia* leaves. A total of 115 phenolic acids were detected in the OL and CL of *C. violifolia* and some of them were also detected in other Brassicaceae plants. Metabolome analysis identified 62 differently regulated phenolic acids, 37 of which were more abundant in the OL and 25 more abundant in the CL. Transcriptome analysis showed that the DEGs were significantly enriched in the phenylpropanoid biosynthesis pathway. Conjoint analysis of transcriptome and metabolome showed that eight genes (*CADH9*, *CYP84A1*, *CYP84A4*, *SGT1*, *CCR2*, *UGT72E1*, and *OMT1*) may participate in the regulation of biosynthesis or accumulation of seven phenolic acids (sinapic acid, sinapyl alcohol, 5-O-caffeoylshikimic acid, sinapoyl malate, coniferin, coniferyl alcohol, L-phenylalanine, and ferulic acid). However, the functions of these genes need further investigation. This work sheds light on the phenolic chemicals found in *C. violifolia* and will encourage its further utilization.

## Figures and Tables

**Table 1:** Annotation statistics of the unigenes in the seven databases.

Database	KEGG	Nr	Swissprot	TrEMBL	KOG	GO	Pfam	Total
Number	60,173	82,706	62,987	82,521	50,137	72,691	57,208	84,140

## Data Availability

The data are available from the first author on reasonable request (raoshen2021@whpu.edu.cn) due to restrictions on sharing data.

## Supplementary Materials

The following supporting information can be downloaded at: https://www.mdpi.com/article/10.3390/metabo12111024/s1, Figure S1: Heatmap (A) and KEGG enrichment (B) analysis of the differentially regulated metabolites; Figure S2: Correlation (A) and principal component (B) analyses between the samples; Figure S3: KOG classification of the differentially expressed genes.
