# Supplementary material for: Revealing the Phenolic Acids in Cardamine violifolia Leaves by Transcriptome and Metabolome Analyses
Source: Metabolites. 2022 Oct 26;12(11):1024. doi: 10.3390/metabo12111024 (PMC9697128; doi:10.3390/metabo12111024)
Supplement: Supplementary file 1 [file metabolites-12-01024-s001.zip › Supplementary Figure S1.pdf]

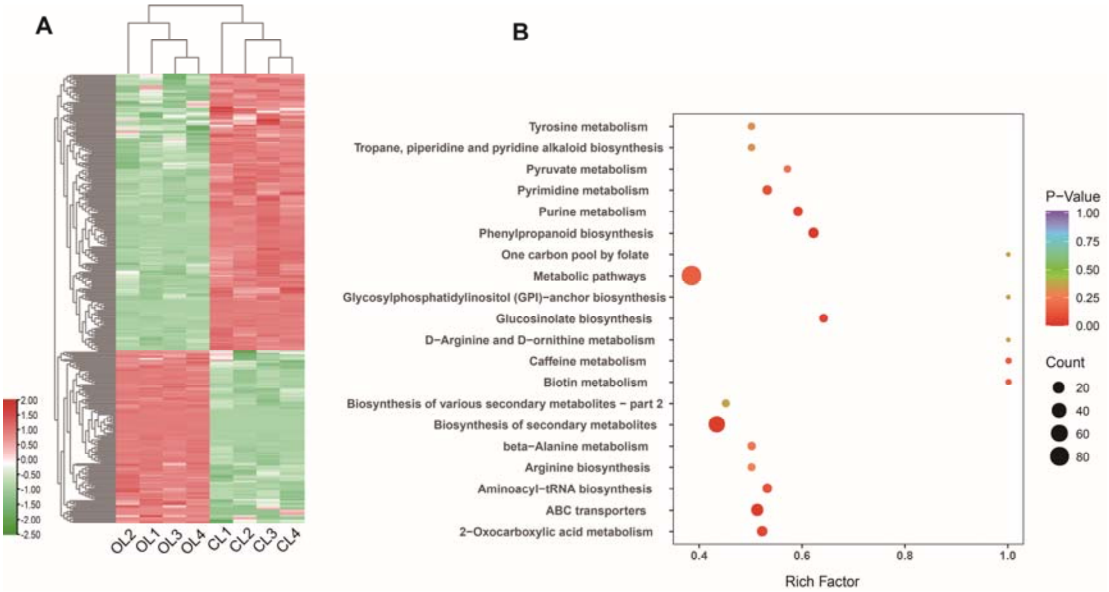

Figure S1 Heatmap (A) and KEGG enrichment (B) analysis of the differentially regulated metabolites
