# Supplementary figures and images for: Revealing the Phenolic Acids in Cardamine violifolia Leaves by Transcriptome and Metabolome Analyses
Source: Metabolites. 2022 Oct 26;12(11):1024. doi: 10.3390/metabo12111024 (PMC9697128; doi:10.3390/metabo12111024)

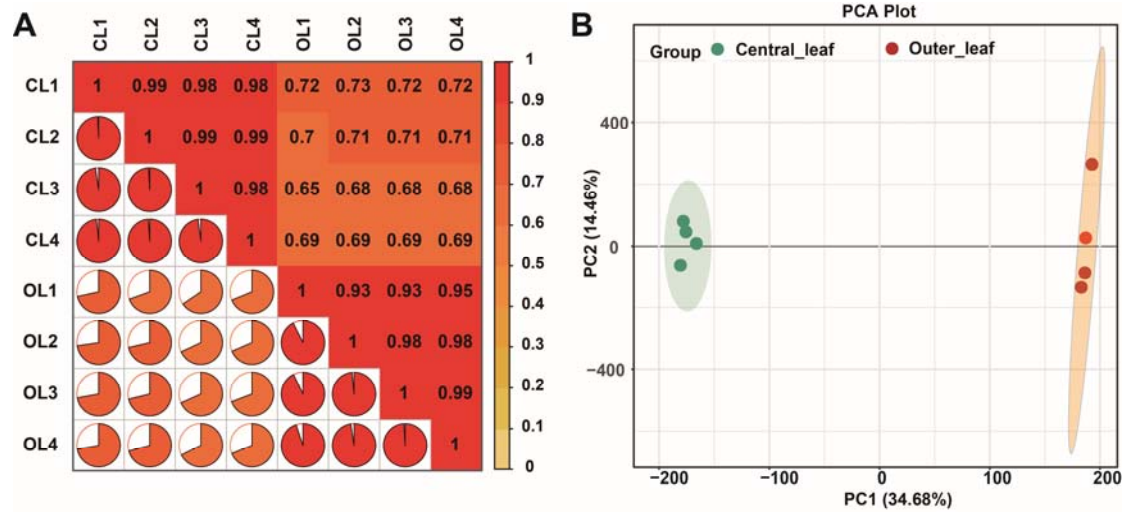

Figure S2 Correlation (A) and principal component (B) analyses between the samples

Supplement: Supplementary file 1 [file metabolites-12-01024-s001.zip › Supplementary Figure S2.pdf]

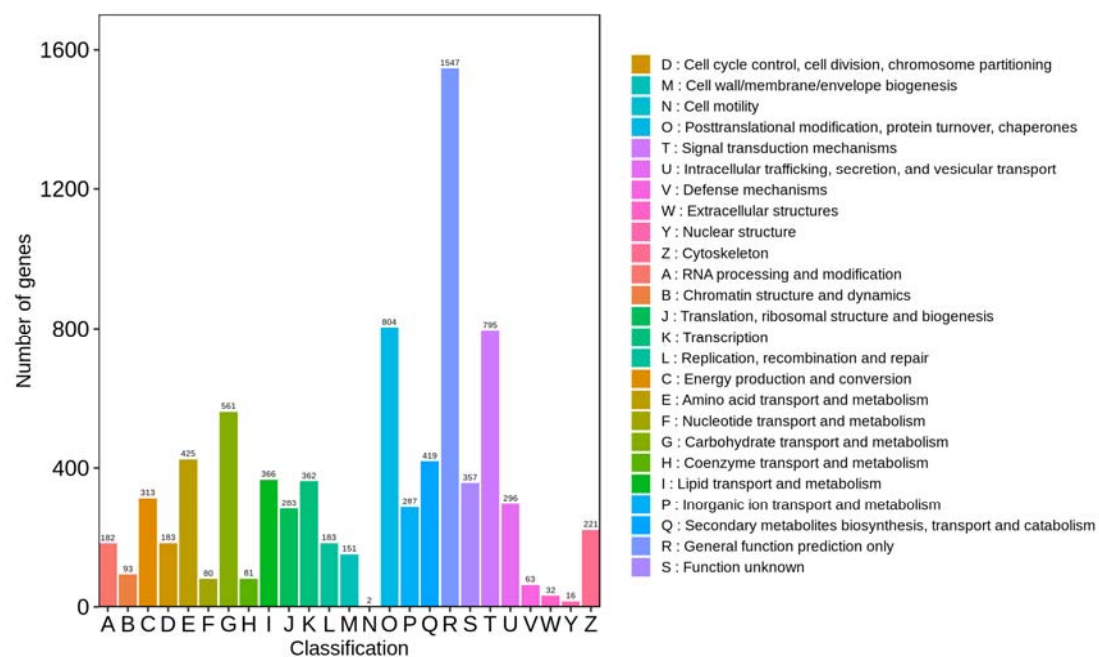

Figure S3 KOG classification of the differentially expressed genes.

Supplement: Supplementary file 1 [file metabolites-12-01024-s001.zip › Supplementary Figure S3.pdf]
